# Cross-sectional study of the rates of military sexual trauma (MST) and associations with adverse mental health outcomes among UK female ex-service personnel: a study protocol

**DOI:** 10.1136/bmjopen-2024-096912

**Published:** 2025-06-26

**Authors:** Tamara Obradovic, Dominic Murphy, Nicola T Fear, Marie-Louise Sharp

**Affiliations:** 1 King’s Centre for Military Health Research, Department of Psychological Medicine, King’s College London, London, UK; 2 Research Department, Combat Stress, Leatherhead, Surrey, UK; 3 Academic Department of Military Mental Health, King’s College London, London, UK; 4 School of Psychology, College of Life and Environmental Sciences, University of Birmingham, Birmingham, England, UK

**Keywords:** Occupational Stress, Adult psychiatry, Anxiety disorders, Eating disorders, Substance misuse, Psychological Stress

## Abstract

**Abstract:**

**Introduction:**

This study investigates the rates of military sexual trauma (MST) and its associations with adverse mental health among a sample of UK female ex-service personnel who served during the Iraq/Afghanistan eras.

**Methods and analysis:**

Female ex-service personnel, who participated in the fourth phase (Phase 4) of the King’s Centre for Military Health Research (KCMHR) Health and Well-being Cohort Study (2022–2023) and consented to be recontacted for follow-up studies (n=295), are being invited to participate in an online questionnaire between July 2024 and February 2025. The questionnaire contains surveys and questions related to experiences of sexual harassment and sexual assault during and outside of military service, disordered eating and broader female health issues. While the questionnaire relates to several female health topics, this study focuses on the surveys related to experiences of sexual trauma and eating disorders. Sociodemographic variables and some health variables, including post-traumatic stress disorder (PTSD), complex PTSD, common mental disorders, alcohol misuse, physical somatisation and social support, will be extracted from participants’ pre-existing data collected in Phase 4 of the KCMHR Cohort Study. Analyses will assess rates of MST, and hierarchical multiple logistic regressions will investigate associated health impacts. Rates and ORs, employing 95% CIs, will be reported.

**Ethics and dissemination:**

This study has been granted full ethical approval by the King’s College London Research Ethics Committee (Ref: HR/DP-23/24–39040). Participants provide informed consent before participating and have access to a signposting booklet containing contact details for a range of support services. A risk protocol is in place, which outlines the procedure to be undertaken if a participant contacts the research team in distress. Findings will form part of a PhD thesis and will be further disseminated through peer-reviewed publication and dissemination with veteran mental health services and charities, and relevant government departments.

**STRENGTHS AND LIMITATIONS OF THIS STUDY**

- The military sexual rauma (MST) measures used in this study ask participants about a wide range of specific behaviours, enabling a more detailed understanding of the rates and mental health associations of different types of MST experiences.
- The sample is restricted to those who served during the recent Iraq and Afghanistan eras, and so findings may not be generalisable to individuals who served during other eras.
- The study is restricted to female ex-service personnel and so cannot provide insight into the experiences or consequences of MST among currently serving female personnel or male serving/ex-service personnel.

## Introduction

 Military sexual trauma (MST) can be conceptualised as experiencing sexually harassing behaviours during military service^1^ and can be associated with long-term health problems in serving and ex-service personnel.^2^ Existing research has found links between MST and mental health conditions, including post-traumatic stress disorder (PTSD), common mental disorders (depression and anxiety) (CMDs),^3^ eating disorders,^4^ substance use disorders^5^ and suicidal ideation.^6^ MST has been associated with negative career outcomes through increasing risk for early separation from military service due to disability, difficulties with work-related activities and demotion.^7^

While sexual trauma in non-military contexts similarly predicts adverse health outcomes,^8^ individuals who report MST often report pre-military experiences of sexual violence.^9 10^ Therefore, understanding potentially cumulative impacts of sexual trauma which occurs during military service requires further research to understand how to mitigate potential adverse outcomes.

Indirect detriments to mental health, via deterred engagement with mental health services and informal support (such as friends and family),^11 12^ further motivates research on sexual trauma which occurs during military service specifically. Although MST can encompass any experience of sexual trauma during military service,^1^ including experiences perpetrated by intimate partners,^13^ some instances of MST occur in occupational settings where the perpetrator is a service member.^14 15^ This may lead to decreased unit and interpersonal support, which has been shown to be associated with PTSD symptoms.^16 17^ Institutional betrayal (defined as when an institution an individual feels dependent on betrays their trust)^18^ may deter engagement with healthcare services perceived to be associated with the military.^19^ Understanding how support services available to ex-service personnel can overcome barriers to engagement in those who have experienced MST is crucial for mitigating adverse health outcomes, particularly if social support resources are affected due to MST experiences.^16 17^

Most research surrounding MST has been conducted in the USA. The unique healthcare services available to US military personnel and veterans in comparison to military personnel and veterans from other countries means that the lack of non-US research inhibits the tailoring of support services in other countries to the needs of individuals with experiences of MST. The understanding of MST in a UK context is limited. While initial UK research has similarly found evidence for sexual assault and sexual harassment during military service predicting harmful alcohol use, PTSD and higher physical somatisation in women ^3^, this study was conducted with women connected with a charity supporting women veterans and restricted to only those who had served in the army, which may not be representative of the wider community (When discussing specific studies, the terms ‘woman’ and ‘female’ are used in accordance with the terminology used by the study authors for consistency. When describing the sample for the study related to this protocol, the term ‘female’ is used to reflect biological sex veterans).

A meta-analysis investigating the prevalence of MST found that serving and ex-service women are more likely to experience MST than serving and ex-service men, with 38.4% of women and 3.9% of men reporting MST.^20^ As well as a disproportionate risk of experiencing MST, a UK review found that female serving and ex-service personnel may face unique barriers to engaging with support services, and hence female serving and ex-service personnel who have experienced MST may experience increased barriers accessing support.^21^ Qualitative research with US ex-service women has illustrated that MST may lead to avoidance of healthcare services due to discomfort in settings such as waiting rooms and concerns surrounding insensitive reactions from healthcare providers.^22^ Health concerns associated with MST which are more likely to affect females than males (such as eating disorders^23^) have been identified as research gaps for UK female serving and ex-service personnel.^24^ As such, understanding the associations between MST experiences and mental health in females within the UK Armed Forces community is needed.

By addressing the limited research surrounding MST in the UK and the unique needs of females in the UK Armed Forces, this study will provide guidance to military, voluntary and National Health Service providers regarding the needs of female ex-service personnel, which may subsequently improve the health outcomes of female ex-service personnel in the UK.

### Aims

1. To assess the rate of MST in UK female ex-service personnel who participated in the fourth phase of the King’s Centre for Military Health Research (KCMHR) cohort study.^25^
2. To investigate the mental health and social support associations of MST in a community sample of UK female ex-service personnel who served during the Iraq/Afghanistan eras.

### Hypotheses

1. Ex-service female personnel who experienced MST, compared with ex-service female personnel who have not experienced MST, will report:
  - Higher rates of adverse mental health.
  - Lower levels of perceived social support.
2. Among ex-service female personnel who experienced MST, high levels of social support (compared with low levels of social support) will be associated with lower rates of adverse mental health.
3. Prior experiences of sexual trauma (pre-military) will exacerbate the associations between MST and adverse mental health and social support.

## Methods and analysis

### Participants

#### Study protocol

##### Sample population and eligibility

This study will recruit participants from the KCMHR Health and Well-being Cohort Study, a longitudinal study exploring the mental health and well-being of UK Armed Forces personnel, regulars and reserves, who served during the Iraq (2003–2011) and Afghanistan (2001–2014) era conflicts. The first wave of data collection for the KCMHR Cohort Study started in June 2004,^26^ with follow-ups taking place in 2007–2009,^27^ 2014–2016^28^ and 2022–2023.^25^ The present study on MST will recruit participants who participated in the fourth wave (Phase 4) of the KCMHR Health and Wellbeing Study.^25^ To be eligible for the study on MST, participants must have: participated in Phase 4 of the KCMHR Health and Well-being Study (2022–2023), consented to be contacted about future research, been identified as female through Defence Statistics, not taken part in other research studies in the department (in addition to Phase 4) within the past 6 months to prevent research fatigue and left the UK Armed Forces. 295 Phase 4 participants meet the eligibility criteria and will be invited to participate.

### Data collection

MST and health data will be collected via an online questionnaire, hosted on Qualtrics,^29^ which takes between 15 and 20 min to complete. Data collection began in July 2024 and is expected to finish in February 2025.

#### Recruitment

##### Initial recruitment attempt

Participants are being contacted via contact details (email, post, text) they have previously provided. Participants with a valid email address are initially contacted via email. Those without an email address are initially sent a link to the survey via text (if mobile phone contact details are available). If neither email nor phone contact details are available, participants are sent a letter containing a QR code and a link to the online questionnaire.

##### Follow-up contact attempts

Individuals who did not respond to the initial recruitment attempt are re-contacted 2–4 weeks later via another mode of contact (email, text or post) and may be re-contacted up to two additional times.

### Participant materials

Potential participants are provided with an online participant information sheet (PIS) and signposting booklet via links embedded in the study invitation. The consent form for this study is built into the landing page of the online survey. Participants are made aware of the potentially distressing elements of the study in the participant invitation, PIS and consent form.

#### Participant information sheet

The PIS explains to participants that participation is voluntary, how they can withdraw from the study, that their data will be handled in line with General Data Protection Regulation (GDPR) requirements, and that their responses are confidential. The PIS provides examples of the questions asked in the survey. Participants are given the opportunity to ask any questions before providing consent and can discuss the project with the study team.

#### Signposting booklet

The signposting booklet provides signposting information for the participant to use in the instance of needing additional support.

#### Survey

Participants are sent the link to an online questionnaire, which contains surveys and items related to female health experiences, disordered eating, sexual function and experiences of sexual assault and harassment (before and after military service) and MST. While this questionnaire contains surveys and items related to broader female health issues and experiences, this study will focus on a specific subset of the surveys included in this questionnaire (experiences of sexual trauma and disordered eating), which are described in further detail below.

### Measures

#### Sociodemographic and military information

Sociodemographic variables will be extracted from pre-existing data collected in the KCMHR Cohort Study. These will include age, marital status, information related to the participant’s military service and information on childhood adversities.

#### Lifetime sexual harassment/sexual assault

Participants’ potential experiences of sexual harassment/sexual assault will be measured using a series of dichotomous, single-item measures which ask participants to indicate whether they have ever experienced sexual harassment or sexual assault: (1) before military service (before the age of 18 years), (2) before military service (after the age of 18 years), (3) during military service, or (4) after military service.

#### MST exposure

Participants’ potential experiences of MST will be measured using:

The US Department of Defense Sexual Experiences Questionnaire (SEQ-DoD),^30^ which assesses participants’ experiences of sexual harassment during military service (including gender harassment, sexual harassment, sexual coercion, unwanted sexual attention and sexual assault) during military service. This measure has been modified for the purposes of the current study to ask participants about MST experiences during their military service, rather than in the last 12 months.

The Online Sexual Harassment Scale (OSHS),^31^ modified to ask specifically about experiences during military service, assesses participants’ experiences of sexual harassment which took place online or on a mobile device while in military service. Table 1 describes the two MST measures.

**Table 1:** Measures of military sexual trauma

Measure	Scoring	Construct(s)	Cut-off for case status
SEQ-DoD^30^	Participants rate the frequency of experiencing behaviours on a 5-point Likert scale (from ‘Never’ to ‘Very often’).	Gender harassment (sexist hostility and sexual hostility), sexual coercion, unwanted sexual attention, sexual assault	Frequency rating >1 of any experience listed in the relevant SEQ-DoD subscales
OSHS(modified)^31^	Participants rate the frequency of experiencing behaviours on a 5-point Likert scale (from ‘Never’ to ‘All the time’).	Online sexual harassment	Frequency rating >1 of any experience listed in the OSHS

OSHS, Online Sexual Harassment Scale; SEQ-DoD, US Department of Defense Sexual Experiences Questionnaire.

#### Health variables

Health outcomes of interest (which include PTSD, complex PTSD (C-PTSD), CMD, alcohol misuse, physical somatisation, disordered eating and social support) will be derived from both pre-existing data collected during Phase 4 and additional survey data collected during this study. Table 2 outlines the outcomes which will be included in analyses.

**Table 2:** Mental health and well-being measures

Construct	Measure(s)	Scoring	Source
Probable PTSD	20-item PCL-5^35^17-item PTSD checklist (PCL-C)^36^	Cut-off for case status: 37/38Cut-off for case status: 49/50	Phase 4
Complex PTSD	International Trauma Questionnaire^37^	Scoring as per^37^	Phase 4
Probable CMD	General Health Questionnaire^38^	Cut-off for case status: 3/4	Phase 4
Alcohol misuse (use of alcohol that is likely to be harmful to health)	Alcohol Use Disorders Identification Test – 10-items^39^	Cut-off for case status: 15/16	Phase 4
Probable eating disorder	Eating Disorder Examination Questionnaire (short)^40^	Cut-off for case status: 14/15^41^	MST follow-up study
Physical somatisation	Patient Health Questionnaire-15^42^	Cut-off for case status: 9/10	Phase 4
Social support	Oslo Social Support Scale^43^	A score of: 8≤indicates poor social support; 9–11 indicates moderate social support; 12–14 indicates strong social support	Phase 4

CMD, common mental disorders; MST, military sexual trauma; PCL-5, PTSD Checklist for DSM-5 (PCL-5); PCL-C, PTSD CheckList – Civilian Version (PCL-C); PTSD, post-traumatic stress disorder.

### Power calculations

Assuming a similar response rate as observed in ex-service females at Phase 4 (60%), a projected sample size of 177 is expected. The statistical package, Stata (V.17),^32^ was used to calculate the power of this sample size to detect changes in the impact of MST exposure on probable PTSD. Power calculations were based on PTSD to enable more conservative estimates due to the low rate of PTSD in ex-service female personnel compared with other mental health outcomes in the Phase 4 sample (11.4%). Assuming that 38.4% of this sample would have been exposed to MST^20^ and adopting a more conservative OR (OR=2.52) of the impact of MST on probable PTSD observed in the literature,^33^ with an alpha of 0.05, the study will have 80% power.

### Planned analyses

#### Analyses

##### Aim 1

The rate of MST in the sample will be calculated by dividing the number of participants who endorse any MST experiences by the number of participants who completed these surveys, multiplied by 100.

##### Aim 2

Analyses will be conducted using Stata (V.17).^32^ In line with Aim 2, analyses will compare the health outcomes of experiencing (vs not experiencing) MST. A series of hierarchical multiple logistic regression models will examine the association between MST experiences and mental health and social support outcomes. ORs, with 95% CIs, will be reported. Age, branch of service, rank, childhood adversities and experiences of sexual harassment or assault before military service (during childhood or as an adult before joining the military) and after leaving military service will be included in the model to control for potential confounding.

### Survey weights

Response weights will be calculated as the inverse probability of responding once sampled for the follow-up study. Response weights will be generated using factors observed previously to predict response (eg, age, rank, regular/reservist status).

### Missing data

Missing items on the key mental health and well-being measures, extracted from the KCMHR dataset were handled according to the procedure outlined in the Phase 4 protocol, where the lowest value was imputed if fewer than four items were missing on the measure.^25^

Missing data from this follow-up study (ie, data related to disordered eating (Eating Disorder Examination Questionnaire (short)) and MST (SEQ-DoD and OSHS)) will be handled using case-wise deletion if rates of missingness are low (ie, less than 5%). If missingness is above 5%, the possibility of multiple imputation will be explored.

### Patient and public involvement

The Phase 4 questionnaire, which will provide some of the outcome measures used for this study, was developed with input from veterans from the Veterans Research Advisory Group, who tested the design and flow of the questionnaire and gave input on outcome measures. Results of the study will be disseminated to participants through social media outlets, the KCMHR website and stakeholder groups such as veteran mental health services and charities.

## Data storage and security

### Database

Personally identifiable information and survey data are stored electronically on a King’s College London secure drive with restricted access. Personally identifiable data and survey data are stored separately. The ‘key’ document linking the ID to personal details will be stored on an encrypted IronKey and on a KCMHR secure drive. Only members of the research team will be able to access this document.

### Pseudonymisation of questionnaire data

Phase 4 participants were assigned a unique ID by the Ministry of Defence when sampled and were assigned a separate unique ID by KCMHR to enable pseudonymous storage of survey data. A third unique ID was assigned to each of the Phase 4 participants meeting the eligibility criteria for this follow-up study, which allows follow-up study data to be held pseudonymously and linked to pre-existing KCMHR Cohort Health and Well-being Cohort Study data.

### Data management and oversight

Pre-existing KCMHR Health and Wellbeing Cohort Study data required for analysis will be extracted from the KCMHR secure system and stored pseudonymously on a King’s College London secure drive. Follow-up study data will be retained for 20 years in line with Medical Research Council (MRC) guidelines, which is stated in the PIS. Data collected in this follow-up study may be shared with other members of the wider KCMHR research team for further analyses. This is stated in the PIS. Members of the research team are provided with training on GDPR, King’s College London standards for handling data and MRC data protection.

## Ethics and dissemination

### Ethical considerations

This study was granted full ethical approval by the King’s College London Research Ethics Committee (Ref: HR/DP-23/24–39040). Participants are informed that they can skip questions they do not wish to answer or can stop the questionnaire if they feel uncomfortable. Participants are given a signposting booklet containing details to relevant support services. Individuals are assigned a unique ID number at the start of the study, ensuring non-identifiable data are held and accessed by the research team.

#### Reimbursement

On completion of the survey, participants are given the opportunity to opt-in to a prize draw to win one of two £50 e-vouchers.

#### Withdrawal procedure

Participants can withdraw their data up until the end of the data collection period. Participants are given the contact details of the research team via the PIS and consent form, whom they may contact if they wish to withdraw. Participants are made aware of the withdrawal terms and procedure on the PIS and consent form

#### Risk protocol

A risk protocol is in place, which outlines the procedure to be followed if participants contact the research team in distress and requires signposting to support services or are in immediate danger.

### Dissemination

Findings will form part of a PhD thesis and will be written up for publication in a peer-reviewed journal. Lay summaries will be shared with key stakeholders, such as veteran mental health services and charities and relevant government departments.

## Discussion

Recent inquiries into serving and ex-service females in the UK Armed Forces suggested that issues of sexual bullying (including harassment and assault) are widespread and have led to calls for policy changes.^34^ This study will provide data to help inform these policies through providing rates of MST and insight into the scope of the problem. Additionally, findings from this study will identify health impacts of MST, which will provide evidence of needs which healthcare providers to veteran populations should address, enabling them to better support those with experiences of MST.
