# Supplementary material for: Cross-sectional study of the rates of military sexual trauma (MST) and associations with adverse mental health outcomes among UK female ex-service personnel: a study protocol
Source: BMJ Open. 2025 Jun 26;15(6):e096912. doi: 10.1136/bmjopen-2024-096912 (PMC12207112; doi:10.1136/bmjopen-2024-096912)
Supplement: Supplementary file 1 [file bmjopen-2024-096912.reviewer_comments.pdf]

PEER REVIEW HISTORY

BMJ Open publishes all reviews undertaken for accepted manuscripts. Reviewers are asked to complete a checklist review form and are provided with free text boxes to elaborate on their assessment. These free text comments are reproduced below.

ARTICLE DETAILS

Title (Provisional)

A Cross-Sectional Study of the Rates of Military Sexual Trauma (MST) and Associations with Adverse Mental Health Outcomes amongst UK Female Ex-Service Personnel: Study Protocol

Authors

Obradovic, Tamara; Murphy, Dominic; Fear, Nicola T; Sharp, Marie-Louise

---

VERSION 1 - REVIEW

**Reviewer**	**1**
**Name**	**Lutgendorf, Monica**
**Affiliation**	**Naval Medical Center San Diego, Obstetrics and Gynecology**
**Date**	**03-Apr-2025**
**COI**	**None**

Thank you for presenting this trial protocol. Overall I think it was very well written and clearly explained. I particularly appreciated your explanation of why this was an important topic to study in the UK military. I also liked your plan to include patient and public involvement in the study. I had two items for you to consider:

1. You are assessing PTSD using the PTSD checklist, I'm curious if you were planning to assess the trauma that participants may have experienced, as individuals may have different trauma experiences, and as you note cumulative trauma is important (perhaps it is already included in the study from which participants are being recruited). One potential way to assess this is the trauma history screen: https://www.ptsd.va.gov/professional/assessment/te-measures/ths.asp

2. Have you considered doing a mixed methods study to further explore in depth participant individual experiences?

Again, thank you for conducting this important work.

**Reviewer**	**2**

**Name**	**Gianoli, Mayumi O.**
**Affiliation**	**VA Connecticut Healthcare System**
**Date**	**27-May-2025**
**COI**	**None**

This is a great proposal for a study. I encourage the following: 1) inclusion of male participants, 2) providing a measure of adverse childhood experiences (ACEs)--and controlling for this in regression analyses, 3) ensure that the measure of lifetime sexual harassment/assault allows for participants to select multiple answers (in case they experienced a particular event before, during and after military). Analyses should contain some sort of regression analyses controlling for pre-military trauma exposure. Research has shown that compared to the rates of ACEs are significantly higher among SMs/Veterans than the general population.

VERSION 1 - AUTHOR RESPONSE

Reviewer 1:

1. You are assessing PTSD using the PTSD checklist, I'm curious if you were planning to assess the trauma that participants may have experienced, as individuals may have different trauma experiences, and as you note cumulative trauma is important (perhaps it is already included in the study from which participants are being recruited). One potential way to assess this is the trauma history screen: https://www.ptsd.va.gov/professional/assessment/te-measures/ths.asp

Thank you for this suggestion. We acknowledge that different trauma experiences, in addition to MST, may contribute cumulatively to subsequent mental health outcomes. However, as this study is using pre-existing PTSD data collected as part of a larger cohort study, it is not possible to implement this suggestion.

2. Have you considered doing a mixed methods study to further explore in depth participant individual experiences?

Thank you for this suggestion. As part of the wider project, we have included a qualitative component involving follow-up interviews with participants who report MST experiences. This will allow us to explore individual experiences in greater depth and provide additional context to the quantitative findings.

Reviewer 2:

1. Inclusion of male participants

Thank you for this suggestion. Whilst we acknowledge that men also experience Military Sexual Trauma (MST), we recognise that there may be differences in experiences, help-seeking behaviours, and subsequent mental health outcomes. This study focuses specifically on the experiences of female ex-service personnel due to potentially unique mental health implications and evidence indicating that women in the UK Armed Forces face unique barriers to accessing support services.

However, it is equally important to study the experiences of male personnel, which should be addressed in a separate, dedicated study.

2. Providing a measure of adverse childhood experiences (ACEs)--and controlling for this in regression analyses

Thank you for this suggestion. Earlier phases of the longitudinal study, in which this study is nested, collected data on childhood adversities. We have updated the analysis plan to incorporate this data into the regression models.

3. Ensur[ing] that the measure of lifetime sexual harassment/assault allows for participants to select multiple answers (in case they experienced a particular event before, during and after military). Analyses should contain some sort of regression analyses controlling for pre-military trauma exposure. Research has shown that compared to the rates of ACEs are significantly higher among SMs/Veterans than the general population.

Thank you for raising this point. The measure used allows participants to indicate whether they experienced sexual harassment and/or sexual assault across multiple time periods (e.g. before, during, and after military service). This data, along with the available information on childhood adversities from earlier phases of the cohort study, will be included in the regression models. We also highlight a change in sampling pool size from 322 to 295. This is due to 27 participants having since been identified as ineligible due to being part of a cohort distinct from the KCMHR Cohort. Implications for projected sample size and study power have been explained in further detail in the manuscript.

We hope that the changes we have made are satisfactory and we look forward to your response.
